# Examining Maternal Cardiometabolic Markers in Pregnancy on Child Emotional and Behavior Trajectories: Using Growth Curve Models on a Cohort Study

**DOI:** 10.1016/j.bpsgos.2023.08.004

**Published:** 2023-08-13

**Authors:** Janell Kwok, Daria P. Khanolainen, Lydia G. Speyer, Aja L. Murray, Minna P. Torppa, Bonnie Auyeung

**Affiliations:** a School of Philosophy, Psychology and Language Sciences, The University of Edinburgh, Edinburgh, United Kingdom; b Department of Teacher Education, University of Jyväskylä, Jyväskylä, Finland; c Department of Psychology, Lancaster University, Lancaster, United Kingdom; d Autism Research Centre, Department of Psychiatry, University of Cambridge, Cambridge, United Kingdom

**Keywords:** Avon ALSPAC, Biomarkers, Child development, Metabolic, Pregnancy

## Abstract

**Background:**

Poor maternal cardiometabolic health in pregnancy is associated with negative effects on child health outcomes, but there is limited literature on child and adolescent socioemotional outcomes. The study aimed to investigate associations between maternal cardiometabolic markers during pregnancy with child and adolescent socioemotional trajectories.

**Methods:**

Growth curve models were run to examine how maternal cardiometabolic markers in pregnancy affected child socioemotional trajectories from ages 4 to 16. Models were adjusted for all pregnancy trimesters and maternal, child, and socioeconomic covariates. This study used the Avon Longitudinal Study of Parents and Children (United Kingdom) cohort. Participants consisted of mother-child pairs (*N* = 15,133). Maternal predictors of fasting glucose, triglycerides, high-density lipoprotein, low-density lipoprotein, and body mass index were taken from each pregnancy trimester (T1, T2, T3). Child outcomes included emotional problems, conduct problems, and hyperactivity problems from the Strengths and Difficulties Questionnaire.

**Results:**

Fully adjusted models showed significant associations between elevated T1 fasting glucose and increased conduct problems, higher T1 body mass index and increased hyperactivity problems, lowered T1 high-density lipoprotein and decreased hyperactivity problems, and elevated T2 triglycerides and increased hyperactivity problems.

**Conclusions:**

Maternal cardiometabolic risk is associated with conduct and hyperactivity outcomes from ages 4 to 16. This study suggests that maternal markers of fasting glucose, low-density lipoprotein, high-density lipoprotein, and triglycerides during pregnancy could be added as supplements for clinical measures of risk when predicting child and adolescent socioemotional trajectories.

Prenatal metabolic markers have been identified in research as modifiable factors predictive of child development outcomes. Maternal adipokines have been linked with placental dysfunction, leading to increased cytokine activity, which contributes to oxidative stress and a toxic environment for the growing fetus (1). Elevated measures of high-density lipoprotein (HDL) and triglycerides from umbilical cord blood at birth are linked with lowered emotion regulation, self-awareness, and interpersonal functioning at age 5 (2). More specifically, in-utero exposure to maternal elevated triglycerides and lowered HDL have been associated with outcomes of cortisol reactivity in 3–5-year-olds, an indicator of stress programming (3). However, there are still literature gaps shown for metabolic adaptations in pregnancy, where disruption of essential biomolecule processes on fetal development is not yet fully understood (4). This study examined how maternal cardiometabolic risk during pregnancy can affect a child’s behavioral and emotional developmental trajectories from childhood to adolescence.

It is suggested that maternal-fetal programming stretches beyond birth outcomes and has an enduring effect on child development. On a clinical scale, maternal obesity and gestational diabetes mellitus have been linked to lowered cognitive skills and psychiatric disorders in offspring (5, 6, 7). Maternal prepregnancy obesity has been linked to inattention symptoms in children aged 5 years (8). This is important as early emotional and behavioral symptoms affect around 20% of children (9,10), presenting further risk factors for lower educational attainment (11,12) and development of psychopathology in adulthood (13,14).

There is also limited evidence on how risk present in different pregnancy trimesters (T1-T3) can potentially affect child development differently. Cohort studies (15) that measured maternal obesity as a risk factor for childhood developmental outcomes looked at either prepregnancy body mass index (BMI) (8,16, 17, 18) or specific trimesters of pregnancy (19). Prenatal inflammatory biological pathways have been shown in third-trimester maternal plasma presenting with risk for later development of autism spectrum disorder (20). As the pregnancy process consists of anti-inflammatory immunologic shifts contributing to fetal development (21), current research is moving in the direction of examining if there are critically vulnerable periods of risk during pregnancy that can potentially lead to future negative childhood outcomes. The study aimed to use available metabolic data from a UK cohort study to examine associations between maternal cardiometabolic risk markers present in all pregnancy trimesters and their effect on childhood emotional and behavioral trajectories from ages 4 to 16 years. These markers include fasting glucose, triglycerides, HDL, low-density lipoprotein (LDL), and BMI. The markers were selected due to availability of data within the UK cohort study and as they are variables usually taken as part of routine clinical visits to aid in future study replicability.

The study hypotheses are 1) maternal cardiometabolic risk during pregnancy is associated with a negative trajectory of emotional and behavioral development from 4 to 16 years old, and 2) maternal cardiometabolic risk applied in mid-to-late pregnancy trimesters affects emotional and behavioral trajectories more than in the earlier pregnancy trimesters.

## Methods and Materials

### ALSPAC Study Participants

This study drew on data from the Avon Longitudinal Study of Parents and Children (ALSPAC) (22). All pregnant women who were residents of Avon (United Kingdom) and expecting a delivery between Apr 1, 1991, and Dec 31, 1992, were eligible for participation and were recruited through maternity clinics, community centers, and advertisements. The final sample used in this study included 15,133 mother-child pairs, with sample sizes dependent on the availability of mother-child pairs when covariates were added to each pregnancy trimester. Maternal cardiometabolic markers were sampled 3 times over pregnancy, and children’s socioemotional and behavioral outcomes were reported on at least 1 time point. Missing data was accounted for in the study analysis.

Ethical approval for the study was obtained from the ALSPAC Ethics and Law Committee and the local research ethics committees, with informed consent for the use of data collected via questionnaires and medical records obtained from participants following the recommendations of the ALSPAC Ethics and Law Committee at the time. Ethics approval was obtained from the Bristol and Weston Health Authority (E1808), and the United Bristol Healthcare Trust (E4445 ALSPAC Focus at Eight). Further information can be found on the ALSPAC study website: http://www.bristol.ac.uk/alspac/researchers/our-data/ or http://www.bristol.ac.uk/alspac/researchers/research-ethics/.

### Cardiometabolic Markers During Pregnancy

Maternal cardiometabolic markers were measured at first (12 weeks), second (18 weeks), and third (32 weeks) trimesters of pregnancy. Information was extracted from maternal clinical obstetric records documented by medical staff. These markers include fasting glucose, triglycerides, HDL, LDL, and BMI. All cholesterol markers were converted to millimoles per liter for comparison in the analysis. Glucose was added as it is the predominant source of energy for the placenta and fetus, as most fetal glucose is derived from the mother (23).

### Social-Emotional and Behavioral Outcomes

Mother self-report measurements from the Strengths and Difficulties Questionnaire (SDQ) (24) were taken as study outcomes. The SDQ was designed to measure childhood socioemotional and behavioral problems, showing high internal consistency and high test-retest reliability across countries. The measure consists of 25 items divided into 5 subscales: emotional problems, hyperactivity, conduct problems, peer relationship problems, and prosocial behavior. This study used subscales of emotional problems, hyperactivity, and conduct problems. Higher scores on these subscales correspond to more adverse socioemotional and behavioral outcomes, with ranges from 0 to 10. Sum scores were calculated for each subscale. SDQ outcomes for ages 4, 7, 8, 9, 11, 13, and 16 were used in this study. For all SDQ outcomes, mother-child pairs participant samples differed as they were based on the availability of data, with no children excluded from analysis.

### Confounders and Covariates

Study confounders included deprivation indices, maternal smoking in the first, second, or third trimester, maternal alcohol intake in the first or third trimester, maternal psychiatric history (bulimia, schizophrenia, anorexia, depression, other recorded psychiatry history), and maternal education. Child covariates included gestation age, birth weight, and child sex. Confounders and covariates were selected based on previous research that has drawn associations with child socioemotional outcomes (25, 26, 27, 28, 29, 30, 31, 32, 33).

### Statistical Analysis

Data was managed in R statistical software (34) and analyzed using Mplus statistical software (https://www.statmodel.com/) (version 8.7). Predictor variables, deprivation, and BMI were mean-centered on, and categorical variables in confounders and covariates were coded so that higher scores represented more risk. Higher SDQ scores implied the presence of more behavioral problems. Structural equation modeling was used to examine how maternal cardiometabolic markers affected child SDQ trajectories over 7 time points (4, 7, 8, 9, 11, 13, and 16 years old).

Analysis was split into a 3-stage process. First, a latent growth curve model was fit for SDQ outcomes. Models were constructed where intercept factors (*i*) represented the intercept of the individual, slope factors (*s*) represented the linear slope of change trajectory, and quadratic factors (*q*) represented the quadratic slope of change trajectory. All loadings for intercept predictor variables started from 0 and were fixed to reflect time spacing for the 7 selected ages in this study. Once latent growth curve models were estimated, the second step was to extend the model to include individual maternal cardiometabolic variables as predictors of latent growth curve components. Nine models were fit, one for each pregnancy trimester per SDQ domain. Last, further analysis was conducted for 3 fully adjusted models where all trimesters were analyzed simultaneously in the same model per SDQ domain. Models were adjusted for confounders and covariates, as stated above. Models were fit using a robust maximum likelihood estimator and evaluated using comparative fit index (CFI), Tucker-Lewis index (TLI), root mean square error of approximation (RMSEA), and standardized root mean square residual (SRMR). Cut-offs for good fit were set at <0.06 for RMSEA and SRMR and ≥0.90 for CFI and TLI (35). For all SDQ outcomes, mother-child pairs participant samples differed as they were based on the availability of data, with no children excluded from analysis. Missing data was handled using full information maximum likelihood estimation. Given the exploratory nature of this study, significance of results was judged based on an alpha level of <0.05; however, we also provide an indicator of significance based on a Bonferroni adjusted *p*-value of .006 (alpha/number of tests [3 trimesters × 3 outcomes = 9 tests]).

## Results

Descriptive statistics showed about one-fourth of the mothers smoked and about half of the mothers consumed alcohol in the first trimester. This percentage decreased slightly by the third trimester, where one-fifth of the mothers smoked and half of the mothers consumed alcohol. Mothers with psychiatric history made up 13.6% of the sample. Deprivation data was missing for half of the cohort, and 14% fell in the lowest (0%–20% quintile) categories. Less than 10% of children had a gestational age of <37 weeks (premature) and <2500 g (low birth weight). Total sample size was 15,133 mother-child participants. More information on descriptive statistics can be found in Tables 1 and 2.

**Table 1:** Study Characteristics

	*n*	%
Child Sex		
Female	7050	48.9%
Male	7372	51.1%
Maternal Smoking (First Trimester)		
Yes	3235	25.4%
No	9504	74.6%
Maternal Smoking (Second Trimester)		
Yes	2342	21.6%
No	8509	78.4%
Maternal Smoking (Third Trimester)		
Yes	2212	19.7%
No	8997	80.3%
Maternal Smoking (Any Trimester)		
Yes	3645	24.3%
No	7243	48.3%
Missing	4121	27.5%
Maternal Alcohol (First Trimester)		
Yes	6835	54.4%
No	5740	45.6%
Maternal Alcohol (Third Trimester)		
Yes	5584	50.1%
No	5571	49.9%
Maternal Psychiatric History		
Yes	1528	13.6%
No	9734	86.4%
Deprivation		
80%–100%	2248	15.0%
60%–80%	1425	9.5%
40%–60%	1325	8.8%
20%–40%	1106	7.4%
0%–20%	1022	6.8%
Missing	7883	52.5%
Child Gestation		
≥37 Weeks	12,584	83.8%
<37 Weeks	1398	9.3%
Missing	1027	6.9%
Child Birth Weight		
≥2500 g	12,488	83.2%
<2500 g	781	5.2%
Missing	1740	11.6%

**Table 2:** Descriptives for Select Variables

Variables	*n*	Mean (SD)	Range
Maternal Metabolic Markers			
First Trimester			
GLU, mmol/L	4495	5.29 (0.98)	2.57–23.14
TRG, mmol/L	4495	1.04 (0.71)	0.24–8.44
LDL, mmol/L	4495	1.47 (0.39)	0.53–3.57
HDL, mmol/L	4495	2.98 (0.82)	0.39–6.62
BMI	5013	26.72 (5.39)	14.58–55.04
Second Trimester			
GLU, mmol/L	2841	5.29 (0.77)	3.46–18.44
TRG, mmol/L	2845	1.03 (0.47)	0.25–5.96
LDL, mmol/L	2845	3.04 (0.74)	0.86–7.17
HDL, mmol/L	2845	1.57 (0.33)	0.36–3.22
BMI	3000	26.36 (5.19)	15.18–54.16
Third Trimester			
GLU, mmol/L	2964	5.26 (0.93)	0.41–20.41
TRG, mmol/L	2964	0.99 (0.50)	0.06–6.41
LDL, mmol/L	2964	3.11 (0.77)	0.91–7.23
HDL, mmol/L	2845	1.58 (0.35)	0.22–4.62
BMI	3126	26.46 (5.29)	16.01–54.24
SDQ			
Conduct Scale			
4 Years	9473	1.96 (1.42)	0–10
7 Years	8421	1.60 (1.46)	0–10
8 Years	7783	1.50 (1.47)	0–10
9 Years	8056	1.28 (1.42)	0–10
11 Years	7346	1.20 (1.42)	0–10
13 Years	7046	1.25 (1.43)	0–10
16 Years	5663	1.02 (1.35)	0–10
Hyperactivity Scale			
4 Years	9479	3.97 (2.33)	0–10
7 Years	8397	3.38 (2.37)	0–10
8 Years	7781	3.34 (2.47)	0–10
9 Years	8056	2.95 (2.26)	0–10
11 Years	7328	2.77 (2.24)	0–10
13 Years	7046	2.91 (2.23)	0–10
16 Years	5663	2.54 (2.12)	0–10
Emotional Scale			
4 Years	9479	1.45 (1.52)	0–10
7 Years	8411	1.51 (1.67)	0–10
8 Years	7780	1.69 (1.83)	0–10
9 Years	8040	1.51 (1.77)	0–10
11 Years	7329	1.46 (1.73)	0–10
13 Years	7049	1.43 (1.71)	0–10
16 Years	5653	1.49 (1.85)	0–10

BMI, body mass index; GLU, fasting glucose; HDL, high-density lipoprotein; LDL, low-density lipoprotein; SDQ, Strengths and Difficulties Questionnaire; TRG, triglycerides.

Model fits were adequate according to fit indices. For fully adjusted models, fit indices for conduct problems showed CFI = 0.978, TLI = 0.964, RMSEA = 0.016, and SRMR = 0.008. Fit indices for hyperactivity problems showed CFI = 0.937, TLI = 0.956, RMSEA = 0.021, and SRMR = 0.009. Fit indices for emotional problems showed CFI = 0.971, TLI = 0.952, RMSEA = 0.016, and SRMR = 0.009. More information can be found in Table S1.

Primary results are first split into individual pregnancy trimesters, with standardized (B*i*) regression coefficients reported to show per unit change of SDQ outcomes for when maternal cardiometabolic risk is present. Each model contained one SDQ domain per pregnancy trimester. Unstandardized values and 95% CIs can be found online in Open Science Framework (titled “Unadjusted models”). The slope (B*s*) and quadratic (B*q*) coefficients are reported to display growth curve trajectories.

The fully adjusted models, including all 3 trimesters, showed some significant effects of metabolic markers of glucose, HDL, BMI, and triglycerides associated with child outcomes. Higher T1 glucose was associated with a steeper increase in conduct problems (B*s* = 0.148) and a slower flattening out (B*q* = −0.146) in the curve over time. Higher T1 HDL was associated with fewer hyperactivity problems (B*i* = −0.080) at age 4. Higher T1 BMI was associated with a steeper increase in hyperactivity problems (B*s* = 0.208) and slower flattening out (B*q* = −0.207) in the curve over time. Higher T2 triglycerides were associated with a steeper increase in hyperactivity problems (B*s* = 0.147) and slower flattening out (B*q* = −0.165) in the curve over time. No individual metabolic markers were associated with the emotional problems domain. Overall, male sex was associated with greater hyperactivity and conduct problems, whereas female sex was associated with greater emotional problems over time. Of note, only T1 glucose remained significant for predicting changes in conduct problems over time after correcting for multiple comparisons. Results on individual trimesters can be found in Table 3, and more information on all models can be found online (https://osf.io/6tpuz/).

**Table 3:** Latent Growth Curve Models of Standardized Intercepts and Slopes of Maternal Metabolic Markers and SDQ Outcomes

Intercept, Slope, and Quadratic	Conduct Scale			Hyperactivity Scale			Emotional Scale		
	T1	T2	T3	T1	T2	T3	T1	T2	T3
Intercept (B*i*) on									
GLU, mmol/L	−0.024 (0.037)	−0.052 (0.038)	0.062 (0.038)	−0.053 (0.035)	0.027 (0.038)	0.027 (0.038)	−0.061 (0.043)	−0.035 (0.047)	0.062 (0.041)
TRG, mmol/L	−0.040 (0.040)	0.042 (0.051)	0.044 (0.053)	0.009 (0.038)	−0.018 (0.047)	0.024 (0.045)	−0.031 (0.040)	0.067 (0.051)	−0.044 (0.051)
HDL, mmol/L	0.055 (0.046)	−0.051 (0.050)	−0.018 (0.048)	−0.080 (0.043)^a^	−0.030 (0.047)	0.006 (0.043)	0.065 (0.044)	−0.084 (0.048)	0.019 (0.040)
LDL, mmol/L	−0.028 (0.041)	0.021 (0.050)	0.023 (0.047)	0.008 (0.039)	0.002 (0.045)	−0.025 (0.043)	0.076 (0.042)	−0.012 (0.051)	−0.044 (0.046)
BMI	−0.046 (0.078)	0.056 (0.137)	0.011 (0.114)	−0.102 (0.071)	0.043 (0.105)	0.085 (0.092)	−0.046 (0.079)	−0.034 (0.090)	0.046 (0.092)
Slope (B*s*) on									
GLU, mmol/L	0.148 (0.048)^b^^,^^c^	−0.032 (0.051)	−0.135 (0.050)^b^	0.051 (0.046)	−0.060 (0.047)	−0.006 (0.050)	0.037 (0.050)	−0.033 (0.064)	0.035 (0.057)
TRG, mmol/L	0.015 (0.054)	0.017 (0.072)	−0.025 (0.069)	−0.051 (0.052)	0.147 (0.067)^a^	−0.085 (0.061)	−0.004 (0.052)	−0.042 (0.065)	0.071 (0.068)
HDL, mmol/L	0.045 (0.059)	0.014 (0.065)	−0.072 (0.055)	0.083 (0.059)	0.107 (0.066)	−0.114 (0.062)	−0.046 (0.056)	−0.034 (0.062)	−0.028 (0.053)
LDL, mmol/L	−0.080 (0.052)	0.010 (0.063)	−0.045 (0.058)	−0.053 (0.054)	−0.055 (0.064)	0.079 (0.057)	−0.060 (0.050)	−0.018 (0.060)	0.014 (0.055)
BMI	0.023 (0.092)	−0.154 (0.128)	0.210 (0.123)	0.181 (0.102)^a^	−0.230 (0.145)	0.099 (0.128)	−0.039 (0.100)	0.030 (0.123)	0.035 (0.116)
Quadratic (B*q*) on									
GLU, mmol/L	−0.146 (0.049)^b^^,^^c^	0.040 (0.057)	0.125 (0.052)^b^	−0.015 (0.052)	0.029 (0.050)	−0.007 (0.053)	−0.018 (0.056)	0.053 (0.065)	−0.053 (0.060)
TRG, mmol/L	0.013 (0.058)	−0.017 (0.076)	−0.025 (0.070)	0.114 (0.057)^a^	−0.165 (0.072)^a^	0.035 (0.068)	0.046 (0.058)	0.067 (0.071)	−0.125 (0.078)
HDL, mmol/L	−0.026 (0.062)	−0.019 (0.071)	0.061 (0.057)	−0.052 (0.061)	−0.080 (0.068)	0.065 (0.061)	0.066 (0.062)	−0.061 (0.068)	0.031 (0.054)
LDL, mmol/L	0.087 (0.054)	−0.013 (0.006)	0.041 (0.060)	0.051 (0.057)	0.040 (0.066)	−0.051 (0.059)	0.027 (0.054)	0.021 (0.065)	0.016 (0.060)
BMI	0.012 (0.094)	0.069 (0.122)	−0.150 (0.121)	−0.183 (0.105)^a^	0.223 (0.140)	−0.085 (0.130)	0.023 (0.107)	0.001 (0.139)	−0.040 (0.126)

Values are presented as estimate (SE). Raw *p* values can be found in Table S5.

BMI, body mass index; GLU, fasting glucose; HDL, high-density lipoprotein; LDL, low-density lipoprotein; T, trimester; TRG, triglycerides.

a *p* < .05 based on raw *p* values.

b *p* < .01 based on raw *p* values.

c Significant based on a Bonferroni-corrected alpha level of .006.

## Discussion

**Figure 1:**
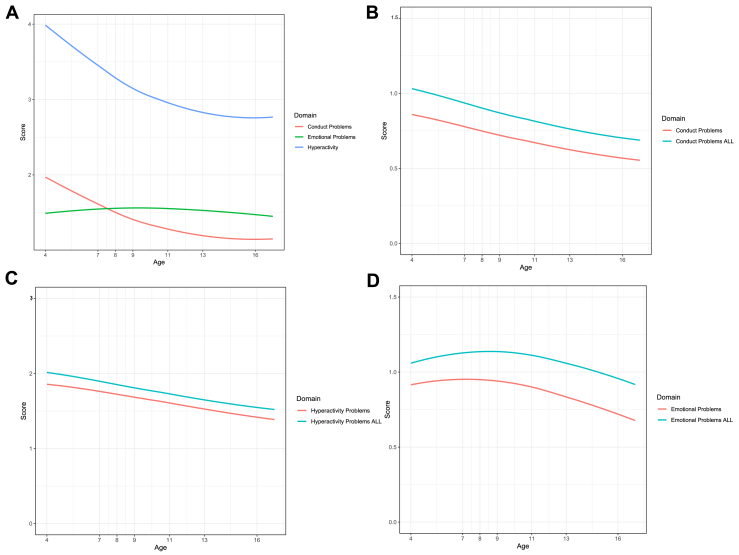
**(A)** Raw plot for Strengths and Difficulties Questionnaire (SDQ) trajectories without adjusting for maternal metabolic risk or covariates^1^. Overall growth curves showing trajectories of SDQ profiles consisting of **(B)** conduct, **(C)** hyperactivity, **(D)** and emotional problems from 4 to 16 years old. Graphs were adjusted for all trimesters. Red curves in **(B–D)** show the SDQ profile of an individual child whose mother had average levels of metabolic markers and average covariates. Blue curves in **(B–D)** show the SDQ profiles of an individual child with whose mother had levels of metabolic markers 2 standard deviations above the mean, to illustrate how developmental trajectories may look for children of individuals with elevated levels of metabolic markers across all trimesters of pregnancy. Metabolic markers included fasting glucose, triglycerides, high-density lipoprotein, low-density lipoprotein, and body mass index.

The purpose of this exploratory study was to examine if maternal cardiometabolic risk during pregnancy was associated with a child’s emotional and behavioral trajectories over time. Study findings supported the first hypothesis and provided a detailed picture of how elevated maternal cardiometabolic risk across pregnancy was associated with a negative change in trajectories for the conduct and hyperactivity domains when compared against an average maternal cardiometabolic profile in pregnancy (Figure 1). Maternal elevated fasting glucose, higher BMI, elevated triglycerides, and lowered HDL showed effects of more problems and slower decrease of problems over time. No associations were found for emotional problems after model adjustments. Study findings also suggested trimester-based effects of maternal metabolic risk, with associations found between trimester one of pregnancy and negative trajectories of children’s conduct and hyperactivity problems. This suggests a window period of vulnerability for when maternal metabolic risk is present in early pregnancy as compared with later pregnancy, not supporting the second hypothesis of the study. Individual markers associated with child development trajectory changes showed some differences across each trimester, particularly in LDL and HDL markers. Mixed results were found for individual biomarkers across each trimester while accounting for covariates, displaying the importance of identifying any critical window of vulnerability during pregnancy for child outcomes.

Study findings further emphasize that pathogenic mechanisms underlying maternal metabolic pathways that influence early life programming are still poorly understood (36). Metabolic conditions can include factors such as nutrition and dietary quality, stress, and inflammation (37). Nonhuman primate studies on maternal inflammation linked obesity with a reduction of serotonin synthesis, suggesting that the same mechanisms are likely responsible for increased risk for behavioral abnormalities or psychological disorders (38). Similarly, maternal inflammation has been found to affect dopaminergic systems and cause an increased risk of offspring with autism spectrum disorder, attention-deficit/hyperactivity disorder, anxiety, and depression (39, 40, 41, 42, 43). To date, literature is still lacking in a complete understanding of how maternal chronic low-grade inflammation, such as metabolic risk, not only disrupts the intrauterine environment (44) but has actual significant effects on fetal programming and future health.

Fetal programming is highly dependent on nutritional availability, coordination, and regulation of biological processes that support pregnancy. Cohort studies on maternal nutrition quality and child emotional and behavioral outcomes showed that a more proinflammatory maternal nutritional profile is linked with childhood outcomes such as increased risk for child emotional problems (45), aggressive behavior symptoms, and even attention-deficit hyperactivity symptoms (46,47). While we did not examine direct markers of exposure, such as maternal diet, the study included biomarkers of health, such as fasting glucose and cholesterol levels, drawing further support for associations with neurodevelopmental outcomes (46). Findings corroborate with research on metabolic flexibility, where more proinflammatory lipid profiles were linked with subsequent childhood health outcomes (48, 49, 50).

A review of evidence has suggested that pregnancy test panels should be expanded to include biomarkers that potentially contribute to inflammation (51). This can not only help to identify disease risks and create targets for intervention during pregnancy (52) but also provide further understanding of fetal and child development. While varying metabolic demands across pregnancy are seen to contribute to fluctuating maternal inflammatory profiles, which affect support for fetal brain development, there are still gaps in understanding of how metabolic adaptations in pregnancy or disruption of essential biomolecule processes affect fetal development (4). Impaired cholesterol homeostasis has been linked with negative embryonic development (4). Maternal hyperlipidemia has been linked with adverse birth outcomes like preterm birth (53), while maternal dyslipidemia in early pregnancy is linked with lowered communication and gross motor skills at 12 months old (54). Despite growing evidence of maternal lipid markers being associated with child outcomes, cholesterol is still commonly not a routine clinical measurement in obstetric practice. Study findings provide further justification for these biomarkers to be included in pregnancy testing panels due to potential long-term effects on child socio-emotional development.

### Implications

Findings demonstrate how maternal cardiometabolic health risk is linked with the growth trajectories of conduct problems and hyperactivity problems across all trimesters, potentially with earlier risk exposure leading to more significant negative outcomes. This study adds to current literature on lowered maternal cardiometabolic health being associated with fetal programming. Findings expand on this by looking beyond physical health and at child emotional and behavioral outcomes from 4 to 16 years old, providing insight into developmental trajectories. Findings also suggest the presence of trimester-based effects when looking at specific markers such as maternal LDL and HDL, showing a need to also explore specific metabolic profiles such as lipid panels during pregnancy.

### Strengths and Limitations

Study strengths include using a large high-quality cohort where metabolic markers were taken from clinical data collected throughout pregnancy trimesters. Self-reports were filled in by parents throughout the ages 4 to 16 years old, allowing for more consistent assessment. This study had several limitations. Due to a lack of data availability across all trimesters, insulin was not included as a predictive biomarker despite literature showing it to be a potential predictor of metabolic and endocrinic disruptions in offspring. The study attempted to supplement this by adding fasting glucose instead, another potential indicator of metabolic and endocrinic disruption. Pubertal growth could have had effects on development due to hormonal changes, which were not accounted for in the study. In addition, this study did not run a full metabolomic analysis panel using logistic regression for risk analysis or examined if any interventions applied to improve cardiometabolic health during pregnancy affected results. Lastly, as with many longitudinal cohort studies, the ALSPAC dataset had a high rate of systemic attrition which may have influenced results in a hard-to-predict direction.

### Conclusions

Maternal cardiometabolic risk during pregnancy is associated with conduct and hyperactivity problems from childhood to adolescence stage. Findings also suggest risk exposure earlier on during pregnancy is associated with more conduct and hyperactivity problems. Findings held after adjusting for all pregnancy trimesters and maternal, child, and deprivation factors. These findings provide new insight into the differential effects of maternal cardiometabolic risk on child and adolescent development, providing justification for more clinical monitoring using these markers. Further research can include expanding clinical services to include metabolic or lipid panels to establish associations between more of these biomarkers and future child developmental trajectories.

## Supplementary Material

Supplementary Data

Key Resources Table

## Footnotes

1 SDQ profile of an individual whose mother had average metabolic health, average socioeconomic status, who did not smoke or drink during pregnancy, and had a baby with gestation ≥37 weeks and birth weight of >2500 g resulted in similar child SDQ profiles when compared with the general population. The raw plot was generated as a comparison model without adjusting for maternal cardiometabolic variables, confounders, or covariates.
