# Supplementary material for: Examining Maternal Cardiometabolic Markers in Pregnancy on Child Emotional and Behavior Trajectories: Using Growth Curve Models on a Cohort Study
Source: Biol Psychiatry Glob Open Sci. 2023 Aug 13;3(4):614–22. doi: 10.1016/j.bpsgos.2023.08.004 (PMC10593919; doi:10.1016/j.bpsgos.2023.08.004)
Supplement: Supplementary Data [file mmc1.pdf]

SUPPLEMENTARY INFORMATION

Examining Maternal Cardiometabolic Markers in Pregnancy on Child Emotional and Behavior Trajectories: Using Growth Curve Models on a Cohort Study

Kwok *et al.*

Table S1. Model Fit Indices

	n	CFI	TLI	RMSEA	SRMR
**T1 CON**	15133	.978	.966	.021	.012
**T1 HYP**	15132	.973	.958	.028	.015
**T1 EMO**	15133	.971	.955	.021	.014
**T2 CON**	15100	.978	.965	.020	.013
**T2 HYP**	15099	.973	.958	.027	.014
**T2 EMO**	15100	.971	.955	.020	.014
**T3 CON**	15101	.978	.964	.019	.011
**T3 HYP**	15100	.973	.957	.026	.013
**T3 EMO**	15101	.971	.954	.019	.013
**All T CON**	15133	.978	.964	.016	.008
**All T HYP**	15132	.973	.956	.021	.009
**All T EMO**	15133	.971	.952	.016	.009

T1: Trimester 1, T2: Trimester 2, T3: Trimester 3, All T: All trimesters adjusted
CON: conduct problems, EMO: emotional problems, HYP: hyperactivity problems

Table S2. R^2 estimates, Intercept, Slope, Quadratic (unadjusted for covariates)

	R^2 Estimates (SE)			Unstandardised Intercepts (SE)			Standardised Intercepts (SE)		
	**I**	**S**	**Q**	**I**	**S**	**Q**	**I**	**S**	**Q**
**Trimester 1**									
Conduct	.004 (.002)	.014 (.006)*	.014 (.007)*	1.970 (.014)***	-1.678 (.053)***	.858 (.053)***	1.807 (.033)***	-.585 (.030)***	.336 (.027)***
Hyperactivity	.010 (.004)**	.008 (.005)*	.006 (.005)	3.984 (.023)***	-2.509 (.080)***	1.292 (.076)***	2.148 (.030)***	-.569 (.027)***	.337 (.024)***
Emotional	.004 (.003)	.007 (.005)	.004 (.004)	1.489 (.016)***	.321 (.065)***	-.359 (.063)***	1.297 (.029)***	.088 (.017)***	-.113 (.019)***
**Trimester 2**									
Conduct	.008 (.005)	.013 (.007)	.014 (.008)	1.969 (.014)***	-1.682 (.053)***	.861 (.053)***	1.806 (.033)***	-.586 (.030)***	.337 (.027)***
Hyperactivity	.006 (.004)	.008 (.006)	.007 (.020)	3.984 (.023)***	-2.512 (.080)***	1.296 (.076)***	2.149 (.030)***	-.570 (.027)***	.339 (.024)***
Emotional	.004 (.003)	.009 (.006)	.005 (.328)	1.490 (.016) ***	.317 (.065)***	-.356 (.063)***	1.297 (.029)***	.086 (.017)***	-.111 (.019)***
**Trimester 3**									
Conduct	.009 (.005)	.021 (.010)*	.019 (.009)*	1.969 (.014)***	-1.681 (.053)***	.861 (.053)***	1.806 (.033)***	-.586 (.030)***	.337 (.027)***
Hyperactivity	.007 (.004)	.001 (.002)	.002 (.003)	2.984 (.023)***	-2.513 (.080)***	1.296 (.076)***	2.149 (.030)***	-.570 (.027)***	.339 (.024)***
Emotional	.001 (.001)	.007 (.006)	.009 (.008)	1.490 (.016)***	.317 (.065)***	-.357 (.063)***	1.297 (.029)***	.086 (.017)***	-.112 (.019)***
**All Trimesters**									
Conduct	.014 (.006)*	.029 (.010)**	.025 (.009)**	1.951 (.016)***	-1.666 (.059)***	.861 (.057)***	1.790 (.034)***	-.581 (.031)***	.337 (.028)***
Hyperactivity	.017 (.005)**	.023 (.010)*	.020 (.010)*	3.957 (.026)***	-2.444 (.088)***	1.254 (.082)***	2.133 (.030)***	-.554 (.028)***	.327 (.025)***
Emotional	.011 (.005)*	.012 (.007)	.014 (.009)	1.480 (.017)***	.299 (.072)***	-.352 (.069)***	1.289 (.030)***	.082 (.019)***	-.111 (.021)***

I: Intercept, S: Slope, Q: Quadratic
p<.05, p<.01**, p<.001****

Table S3. R^2 estimates, Intercept, Slope, Quadratic (adjusted for covariates)

	**R^2 Estimates (SE)**			**Unstandardised Intercepts (SE)**			**Standardised Intercepts (SE)**		
	**I**	**S**	**Q**	**I**	**S**	**Q**	**I**	**S**	**Q**
**Trimester 1**									
Conduct	.052 (.007)***	.017 (.007)*	.017 (.007)*	1.005 (.158)*	-.875 (.609)	-.042 (.565)	.754 (.265)**	-.463 (.369)	.075 (.376)
Hyperactivity	.040 (.006)***	.015 (.006)*	.018 (.007)	3.478 (.259)*	-1.282 (.975)	-.717 (.897)	1.96 (.250)***	-.812* (.358)	.229 (.379)
Emotional	.017 (.004)***	.018 (.007)**	.015 (.007)*	1.062 (.181)*	1.077 (.792)	-1.703 (.783)	1.18 (.277)***	.238 (.360)	-.627 (.402)
**Trimester 2**									
Conduct	.057 (.008)***	.015 (.008)*	.017 (.008)*	1.008 (.161)*	-1.007 (.620)	.077 (.575)	.829 (.320)*	-.051 (.438)	-.245 (.454)
Hyperactivity	.038 (.006)***	.015 (.007)*	.019 (.008)*	3.377 (.261)	-1.203 (.984)	-.811 (.902)	1.72 (.321)***	-.395 (.428)	-.037 (.439)
Emotional	.017 (.005)***	.018 (.007)*	.015 (.007)*	1.034 (.183)	1.244 (.804)	-1.848 (.795)	1.40 (.335)***	.101 (.434)	-.383 (.481)
**Trimester 3**									
Conduct	.058 (.008)***	.027 (.010)**	.024 (.010)*	.969 (.162)	-1.072 (.628)	.178 (.583)	.561 (.297)	.320 (.398)	-.655 (.403)
Hyperactivity	.039 (.006)***	.010 (.005)*	.015 (.006)*	3.403 (.267)	-1.454 (.996)	-.584 (.913)	1.48 (.289)***	-.255 (.418)	-.142 (.434)
Emotional	.014 (.004)***	.017 (.007)*	.020 (.009)*	1.069 (.186)	1.058 (.821)	-1.753 (.812)	1.05 (.307)**	.320 (.398)	-.591 (.442)
**All Trimesters**									
Conduct	.062 (.008)***	.035 (.010)**	.030 (.010)**	.519 (.620)***	-2.596 (-2.209)	-1.415 (-1.055)	.859 (.151)***	-.340 (.220)	.035 (.229)
Hyperactivity	.047 (.007)***	.032 (.011)**	.033 (.011)**	3.451 (.266)***	-1.577 (.998)	-.433 (.917)	1.856 (.146)***	-.357 (.227)	-.113 (.238)
Emotional	.024 (.006)***	.023 (.008)**	.024 (.010)*	1.051 (.187)***	1.015 (.816)	-1.639 (.811)*	.915 (.164)***	.277 (.223)	-.515 (.255)*

I: Intercept, S: Slope, Q: Quadratic
p<.05, p<.01**, p<.001**

Table S4. Correlations table for maternal metabolic markers, confounders, and covariates

	T1	T2	T3	T1 Maternal Smoking	T2 Maternal Smoking	T3 Maternal Smoking	T1 Maternal Alcohol	T3 Maternal Alcohol	Maternal Psychiatric History	Deprivation	Gestation	Birthweight
**T1**												
**GLU**	-	.709	.710	.056	.071	.072	.000	-.007	-.001	.048	-.022	-.015
**BMI**	-	.939	.931	.040	.039	.036	-.022	-.052	-.010	.159	.008	-.003
**LDL**	-	.756	.734	.054	.049	.052	.002	-.007	.014	.031	.016	.004
**HDL**	-	.818	.771	-.144	-.161	-.154	.063	.152	-.002	-.137	-.033	-.014
**TRG**	-	.752	.737	.153	.173	.169	.008	-.011	.085	.087	.016	.000
**T2**												
**GLU**	.709	-	.698	.071	.085	.089	.020	.002	-.009	.042	-.012	-.003
**BMI**	.939	-	.960	.053	.053	.040	-.024	-.067	.003	.167	.003	-.008
**LDL**	.756	-	.800	.024	.045	.036	-.002	.043	.029	-.024	.018	.010
**HDL**	.818	-	.801	-.121	-.125	-.120	.068	.144	.012	-.143	-.029	-.009
**TRG**	.752	-	.796	.159	.167	.160	-.005	-.030	.081	.104	.005	-.014
**T3**												
**GLU**	.710	.698	-	.073	.066	.078	.026	.011	-.014	.044	-.018	.009
**BMI**	.931	.960	-	.048	.051	.040	-.023	-.066	.000	.169	.007	-.004
**LDL**	.734	.800	-	.015	.022	.017	.004	.033	.006	-.019	.026	.026
**HDL**	.771	.801	-	-.110	-.127	-.124	.076	.149	-.003	-.138	-.014	.015
**TRG**	.737	.796	-	.154	.161	.165	-.002	-.014	.071	.116	.014	-.003

T1: Trimester 1 corresponding marker, T2= Trimester 2 corresponding marker, T3= Trimester 3 corresponding marker
p<.05, p<.01**, p<.001****
**Data on maternal alcohol intake for second trimester not available*

Table S5. Raw p-values.

P-values	Conduct T1 Raw p-values	Conduct T2 Raw p-values	Conduct T3 Raw p-values	Hyperactivity T1 Raw p-values	Hyperactivity T2 Raw p-values	Hyperactivity T3 Raw p-values	Emotional T1 Raw p-values	Emotional T2 Raw p-values	Emotional T3 Raw p-values
**Intercept (B*i*) on**									
Glucose	.504	.165	.100	.132	.482	.469	.152	.459	.132
Triglycerides	.319	.407	.402	.818	.705	.594	.447	.187	.386
HDL	.225	.310	.709	.064	.519	.124	.142	.081	.641
LDL	.499	.669	.631	.833	.97	.564	.072	.809	.333
BMI	.558	.684	.921	.151	.680	.360	.560	.701	.619
**Slope (B*s*) on**									
Glucose	.002^{+}	.532	.007	.277	.197	.903	.462	.612	.538
Triglycerides	.779	.817	.715	.323	.031	.167	.945	.523	.297
HDL	.449	.831	.192	.159	.104	.066	.415	.588	.597
LDL	.122	.877	.438	.328	.390	.168	.238	.768	.797
BMI	.806	.232	.088	.075	.113	.437	.693	.807	.764
**Quadratic (B*q*) on**									
Glucose	.003^{+}	.479	.017	.777	.023	.608	.750	.413	.383
Triglycerides	.821	.821	.723	.046	.239	.285	.429	.342	.111
HDL	.670	.787	.282	.391	.541	.384	.290	.368	.575
LDL	.107	.846	.494	.083	.110	.513	.622	.749	.790
BMI	.899	.573	.215	.565	.898	.103	.826	.995	.749

$^{+}$*significant based on a Bonferroni corrected alpha level of .006*
